# All Purkinje cells are not created equal

**DOI:** 10.7554/eLife.03285

**Published:** 2014-06-10

**Authors:** Catarina Albergaria, Megan R Carey

**Affiliations:** 1 **Catarina Albergaria** is in the Champalimaud Neuroscience Programme, Champalimaud Centre for the Unknown, Lisbon, Portugal; 2 **Megan R Carey** is in the Champalimaud Neuroscience Programme, Champalimaud Centre for the Unknown, Lisbon, Portugalmegan.carey@neuro.fchampalimaud.org

**Keywords:** cerebellum, cerebellar modules, Purkinje cells, zebrin II, TRPC3, neural circuits, mouse

## Abstract

Although the wiring of the cerebellar cortex appears to be uniform, the neurons in this region of the brain behave more differently from each other than previously thought.

**Related research article** Zhou H, Lin Z, Voges K, Ju C, Gao Z, Bosman LWJ, Ruigrok TJH, Hoebeek FE, De Zeeuw CI, Schonewille M. 2014. Cerebellar modules operate at different frequencies. *eLife* **3**:e02536. doi: 10.7554/eLife.02536

**Image** Purkinje cells in the cerebellar cortex show a striped pattern of zebrin expression (in green)

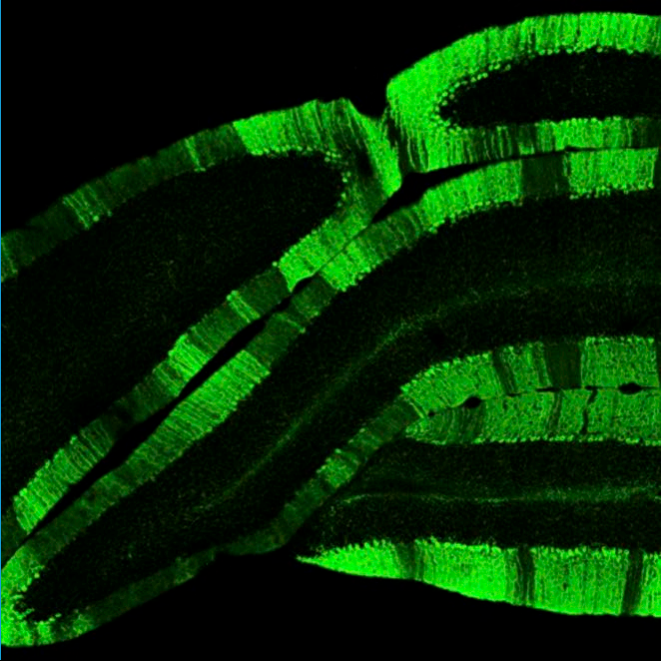

How can the activity in our brains give rise to our thoughts, movements and experience of the world around us? An obvious first step in any attempt to answer this question is to understand how neurons are ‘wired’ together to support these functions. The cerebellum, or ‘little brain’, is located at the back of the brain and is important for coordinated motor control and learning. While different regions of the cerebellum are connected to different parts of the brain, the pattern of wiring within the cerebellar cortex (the outer layer of the cerebellum; Figure 1) is highly consistent.

**Figure 1.**
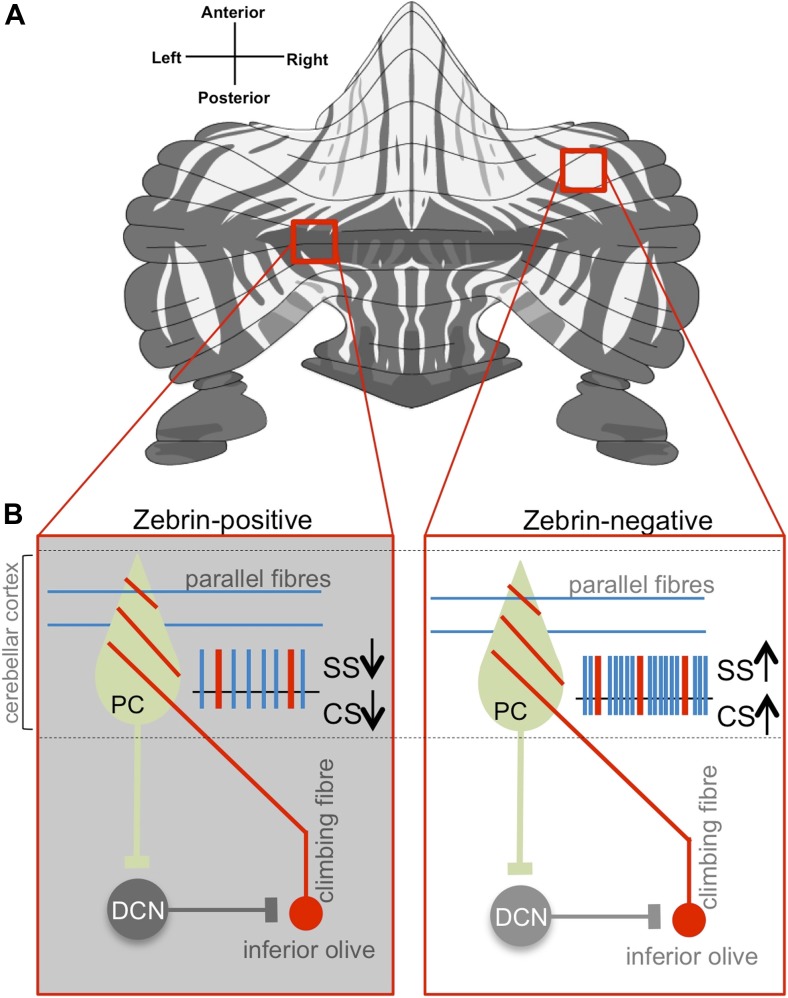
Purkinje cell activity differs based on zebrin identity. (**A**) The alternation of zebrin-positive (dark) and zebrin-negative (light) zones gives the cerebellum a striped pattern. (**B**) Purkinje cells (PC) are the only neurons that carry signals out of the cerebellar cortex; they receive input signals from thousands of parallel fibres (blue) and one single climbing fibre (red). Climbing fibres arise from a region of the brainstem called the inferior olive. The inputs from parallel fibres (integrated with other inputs, not shown) lead to high-frequency simple spikes (SS, blue) in the Purkinje cell. The climbing fibre produces infrequent complex spikes (CS, red). Purkinje cells inhibit neurons in the deep cerebellar nuclei (DCN), which in turn inhibit neurons in the inferior olive. Zhou, Lin et al. found that both simple and complex spike firing frequency of Purkinje cells throughout the cerebellar cortex was decreased in zebrin-positive zones (shown on the left) when compared to zebrin-negative zones (shown on the right).

FIGURE CREDIT: Zebrin map modified from Sugihara and Quy, 2007

This consistency, combined with the fact that all nerve impulses leaving this region are carried by cells of a single type—Purkinje cells—has raised the possibility that the same circuit computation could underlie all cerebellar functions (Bloedel, 1992).

Now, in *eLife*, Martijn Schonewille, Chris De Zeeuw and co-workers at the Erasmus University Medical Center—including Haibo Zhou and Zhanmin Lin as joint first authors—challenge this notion by showing that Purkinje cells behave in different ways (Zhou et al., 2014), and that these differences map onto well-established patterns of gene expression.

Previous work has shown that the cerebellum is organized into genetically defined subdivisions (Apps and Hawkes, 2009; Hawkes, 2014). There is a patterned expression of multiple molecular markers within the cerebellar cortex, with zebrin II being of major interest because it is highly conserved across vertebrates. Staining for zebrin II reveals dramatic striped patterns of zebrin-positive (‘Z+’) and zebrin-negative (‘Z−‘) Purkinje cells (Figure 1; Sugihara and Quy, 2007).

Z+ and Z− zones could represent distinct functional units within the cerebellum (Apps and Hawkes, 2009; Ruigrok, 2011; Hawkes, 2014). Purkinje cells within individual modules receive inputs from, and send output to, distinct populations of neurons (Oscarsson, 1979). In addition, Z+ and Z− Purkinje cells have different propensities for changing their connections with other neurons (a process that is thought to underlie cerebellum-dependent motor learning: Wadiche and Jahr, 2005; Ebner et al., 2012). Nevertheless, until now, the physiological properties of Z+ and Z− neurons have not been compared systematically, and many researchers have treated Purkinje cells more or less interchangeably.

Now Schonewille, De Zeeuw—who is also at the Netherlands Institute for Neuroscience—and co-workers have recorded the electrical activity from nearly 250 Purkinje cells in awake mice. Neurons were sampled from throughout the cerebellar cortex, and recording sites were marked with dye so that many of the same neurons could be identified following the recordings as either Z+ or Z−. Purkinje cells show two distinct kinds of nerve impulses (called ‘simple spikes’ and ‘complex spikes’), each driven by inputs from a different kind of nerve fibre (Figure 1). Zhou, Lin et al. found that both simple and complex spikes ‘fire’ more frequently in Z−, compared to Z+, Purkinje cells.

However, since Z+ and Z− Purkinje cells are more likely to be found in different parts of the cerebellar cortex, the variations in firing rates might be a consequence of the neurons' different locations, and not directly associated with zebrin identity per se. To test this idea, Zhou, Lin et al. performed an additional, technically challenging, imaging experiment in living mice that were genetically engineered to express a green fluorescent protein in a zebrin-like pattern. Even when recordings were taken from adjacent Z+ and Z− Purkinje cells in individual experiments, the Z− cells still fired more frequently than the neighbouring Z+ cells. This suggests that the differences in physiological properties are in fact correlated with zebrin identity.

What causes the observed differences between Z+ and Z− Purkinje cells is still not completely clear. Zhou, Lin et al. ruled out a direct role for the enzymatic activity of zebrin. They also provided evidence that the differences could result from properties intrinsic to Purkinje cells themselves, rather than the inputs that Purkinje cells receive from different nerve fibres. Zhou, Lin et al. found that manipulating TRPC3—an ion channel that works with other proteins that are expressed in zebrin-negative bands—did alter some of the physiological properties that differed between Z+ and Z− Purkinje cells. TRPC3 itself, however, does not appear to be expressed in a striped pattern, and direct links between zebrin, TRPC3, and simple and complex spikes remain speculative.

Might there be some other pattern that determines the physiological properties of neurons lurking, as yet undiscovered, within the cerebellar cortex? Many other molecules exhibit striped expression patterns (Hawkes, 2014). Some share boundaries with zebrin stripes, but others form their own patterns, sometimes subdividing Z+ areas. Intriguingly, Zhou, Lin et al. observed that, beyond the zebrin-dependent differences, the physiological properties of Z+ Purkinje cells vary systematically across cerebellar regions. This suggests that something besides the zebrin map might influence these neurons’ physiological properties.

Thus, the findings of Zhou, Lin et al. raise the possibility that multiple molecular maps with their own distinct physiological profiles could co-exist within the cerebellar cortex. Taken together with the presence of tightly regulated loops between Purkinje cells and their inputs and outputs (Figure 1), the potential implications are far-reaching. For instance, specific behaviours could be governed by independent, functionally distinct modules. If borne out by future studies, this could imply a dizzying complexity of functional diversity for a circuit that is so often touted as being ‘simple and well-understood’. Finally, it is an important reminder that the function of even seemingly identical neural circuits can be modulated by differences in their intrinsic properties that change their capability to process information (Bargmann and Marder, 2013).
